# Metastasis to Breast From Carcinoma Gallbladder: A Case Report and Review of Literature

**DOI:** 10.7759/cureus.11307

**Published:** 2020-11-03

**Authors:** Bijayalaxmi Sahoo, Sandip Barik, Pritinanda Mishra, Saroj Kumar Das Majumdar, Dillip Kumar Parida

**Affiliations:** 1 Radiation Oncology, All India Institute of Medical Sciences, Bhubaneswar, IND; 2 Pathology, All India Institute of Medical Sciences, Bhubaneswar, IND

**Keywords:** breast metastasis, gall bladder cancer, uncommon site, metastatic gall bladder, chemotherapy, survival

## Abstract

Gallbladder cancer (GBC) is the commonest malignancy among biliary tract cancers. Locoregional spread in GBC is more common than distant metastasis. The liver and abdominal lymph nodes is the most common site of distant metastasis. Breast metastasis is a rare site of dissemination. GBC is an aggressive tumor and carries a poor prognosis, with a five-year survival rate of less than 10%. Metastasis to the breast from a gallbladder is significantly less and accounts for very few cases. Here, we are reporting a rare case of carcinoma gallbladder metastasis to the breast who survived for 38 months from the diagnosis of GBC and around 25 months after breast metastasis.

## Introduction

Gallbladder cancer (GBC) is the most common malignancy of the biliary tract, representing 80%-95% of biliary tract cancers worldwide. It is the sixth most common cancer among gastrointestinal (GI) cancer [1]. It accounts for only 2% to 4% of all malignant GI tumors [2-3]. Cholelithiasis and chronic cholecystitis are the most common predisposing factors associated with GBC. Women in the seventh decade are affected three to four times more often than men [4]. Locoregional spread in GBC is more common than distant metastasis. Distant metastases usually occur in the liver, lymph nodes, and peritoneum [5]. GBC is an aggressive tumor and carries a poor prognosis with a five-year survival rate of less than 10% (1). GBC metastasis to the breast is very rare. To date, as per our knowledge, only three cases have been reported [6].

 We report a case of carcinoma gallbladder with breast metastasis and a short review of the literature.

## Case presentation

A 55-year-old female, known case of hypothyroidism, on medication for five years, presented with complaints of a painless lump over an old abdominal scar for one month. The patient had undergone laparoscopic cholecystectomy in 2015 for chronic cholelithiasis. The histopathological study of the operated specimen was suggestive of biliary intraepithelial neoplasm. On examination, a 2.5 x 2 cm, hard, non-tender nodule was palpable over the cholecystectomy scar. Fine needle aspiration cytology (FNAC) was advised from the scar nodule, which showed metastatic adenocarcinoma. A contrast-enhanced computed tomography (CT) scan showed a solid mass lesion of size 3x2 cm in the parietal wall of the anterior abdominal in the epigastric region on the right side, with infiltration to the right rectus sheath and muscle, along with enlarged periportal and para-aortic lymph nodes. Her CA 19.9 level was elevated (3.73 ng/ml).

She was started on gemcitabine (1 gm/m^2^) and oxaliplatin (100 mg/m^2^) based chemotherapy. She defaulted treatment after completing three cycles of chemotherapy. Contrast-enhanced computed tomography (CECT) abdomen done five months after the discontinuation of treatment showed an increase in lesion size for which she underwent wide local excision of the abdominal wall nodule. The histopathology report showed metastatic adenocarcinoma with a tumor size of 6 x 5 x 4 cm. She was continued gemcitabine (1 gm/m^2^) and oxaliplatin (100 mg/m^2^) chemotherapy for three more cycles. After completing three cycles of chemotherapy, she was on regular follow-up.

**Figure 1:**
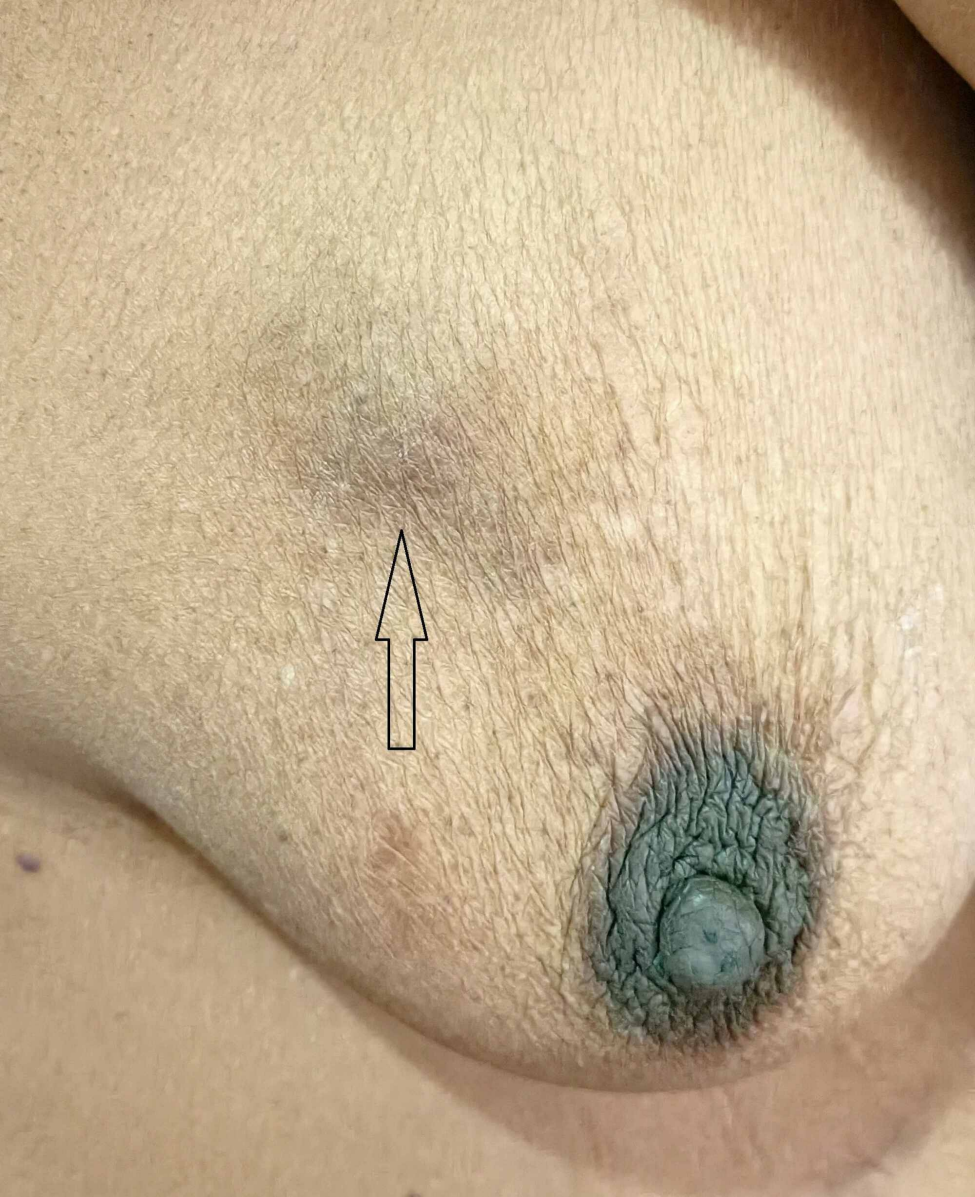
Hard lump sized 3 x 1.5 cm over the UOQ of the right breast UOQ: upper outer quadrant

After one year of completing chemotherapy, the patient developed a right side breast lump. On examination, a hard, non-tender lump of size 3 x 1.5 cm palpable over the upper outer quadrant (UOQ) of the right breast with no axillary lymphadenopathy (Figure 1).

**Figure 2:**
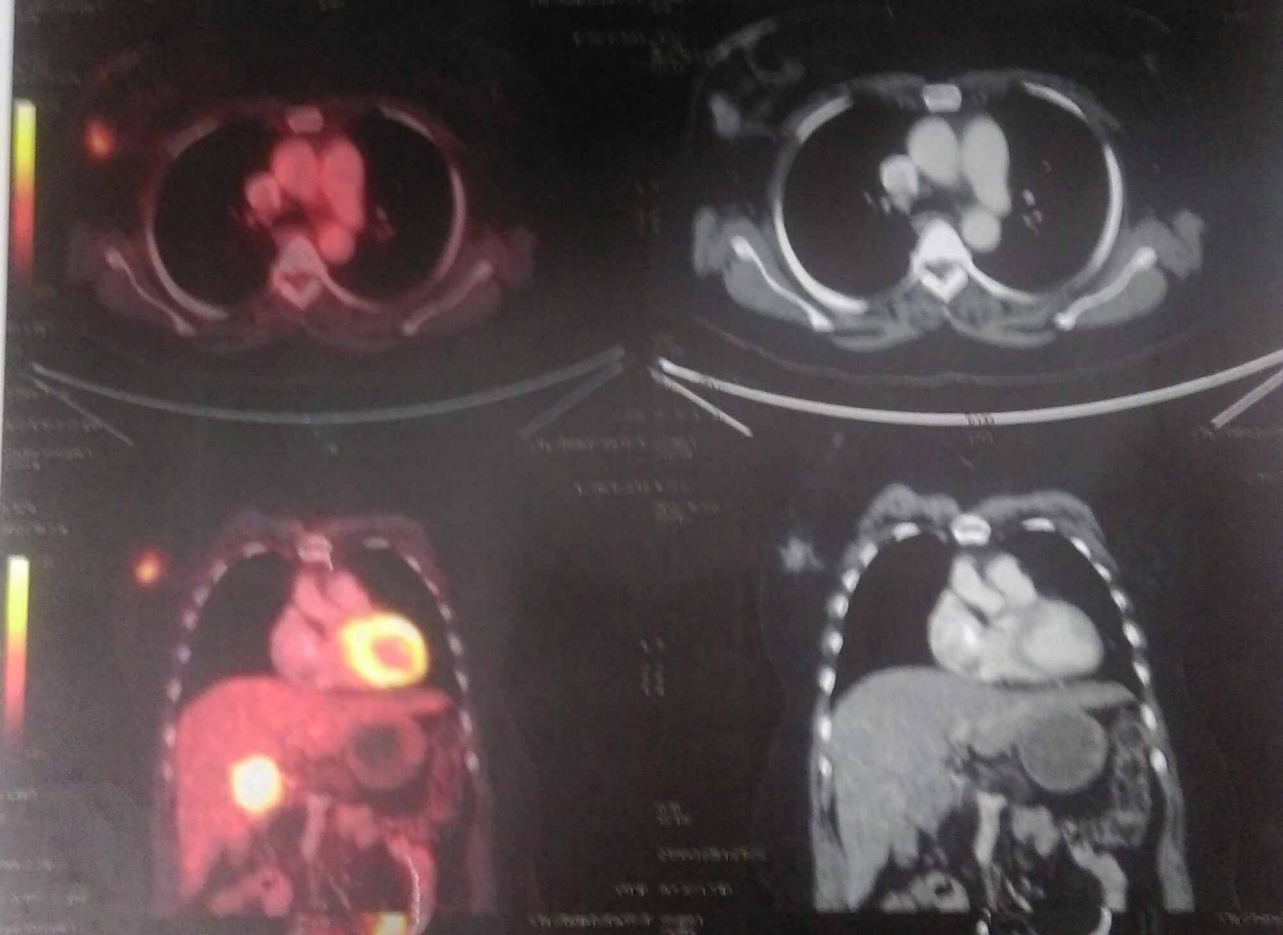
Whole-body PET-CT scan showing increased FDG uptake in the UOQ of the right breast of size 1.9 x 2.7 cm, SUVmax = 3.7 PET-CT: positron emission tomography-computed tomography; FDG: fluorodeoxyglucose; UOQ: upper outer quadrant; SUVmax: maximum standard unit value

The positron emission tomography-computed tomography (PET-CT) scan showed increased uptake of fluorodeoxyglucose (FDG) in the gallbladder fossa and UOQ of the right breast of size 1.9 x 1.7 cm, with a standardized uptake value (SUV) of 3.7 (Figure 2).

**Figure 3:**
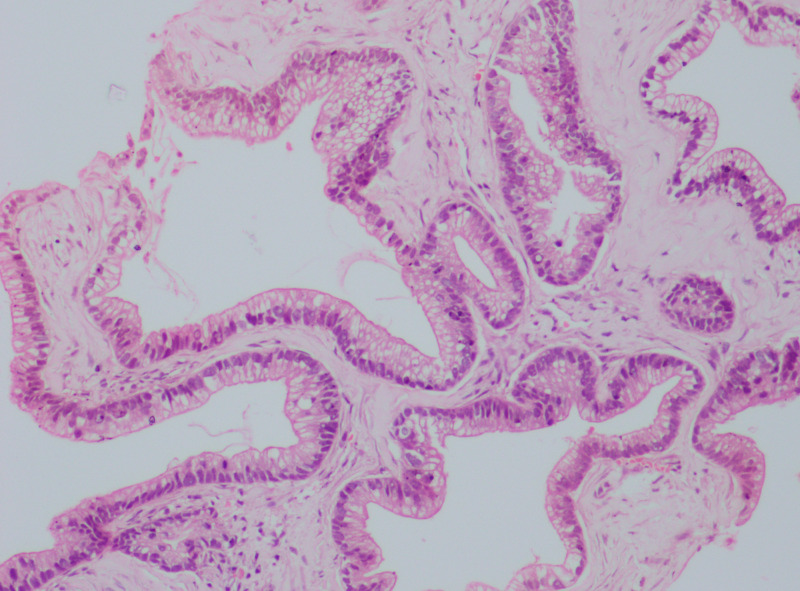
10x H&E microscope feature Showing the cells have elongated nuclei and supra-nuclear clearing, so metastatic adenocarcinoma possibly from gallbladder primary H&E: hematoxylin and eosin

**Figure 4:**
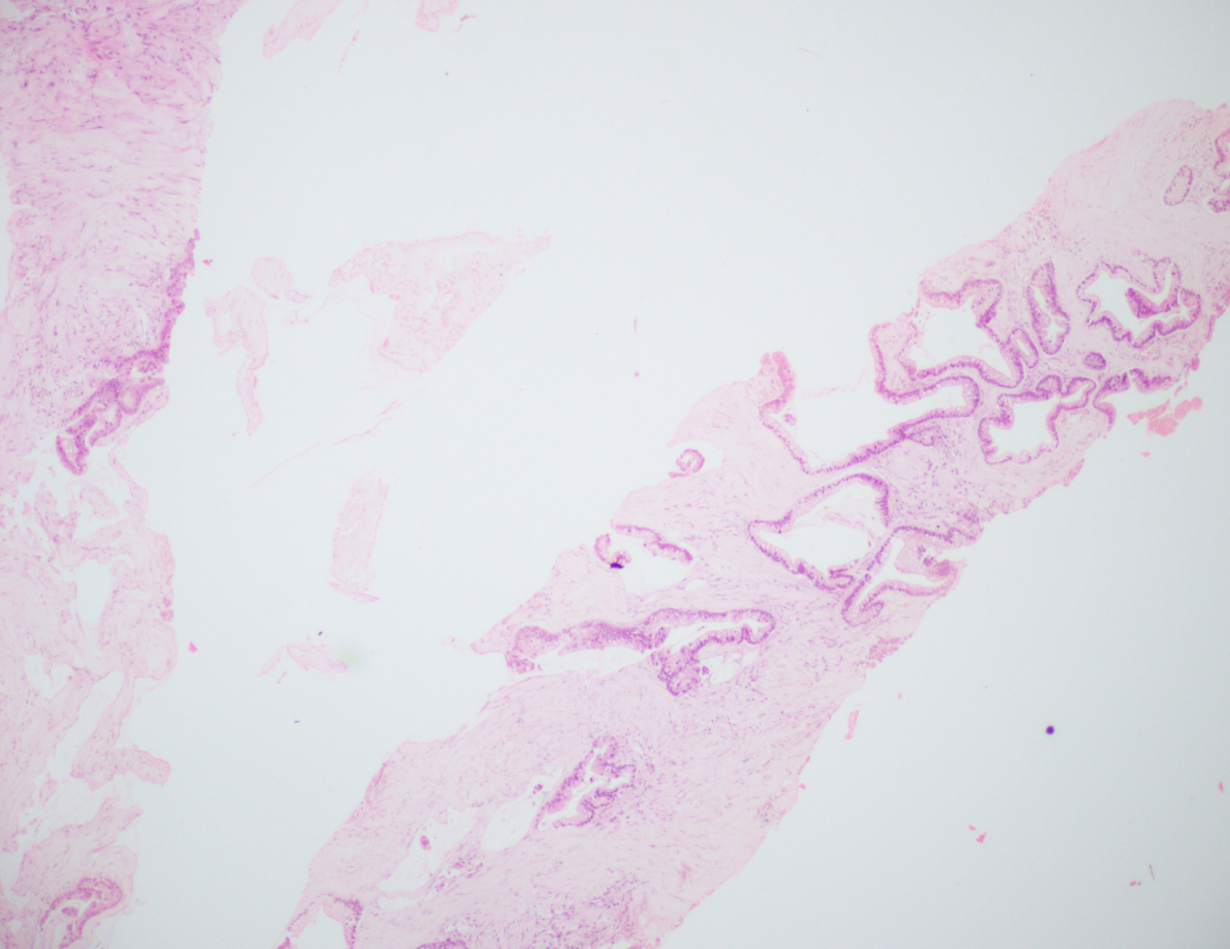
2x H&E microscope view from the breast lump Showing linear cores of fibrocollagenous tissue with malignant epithelial cells in a glandular pattern

Excisional biopsy of the breast lump showed metastatic adenocarcinoma with CK-7 and CK-20 strongly positive. CDX-2, GATA-3, mammaglobulin, estrogen receptor (ER), progesterone receptor (PR), and human epidermal growth factor receptor 2 (HER2/neu) was negative, compatible with the primary biliary origin (Figures 3-4).

**Figure 5:**
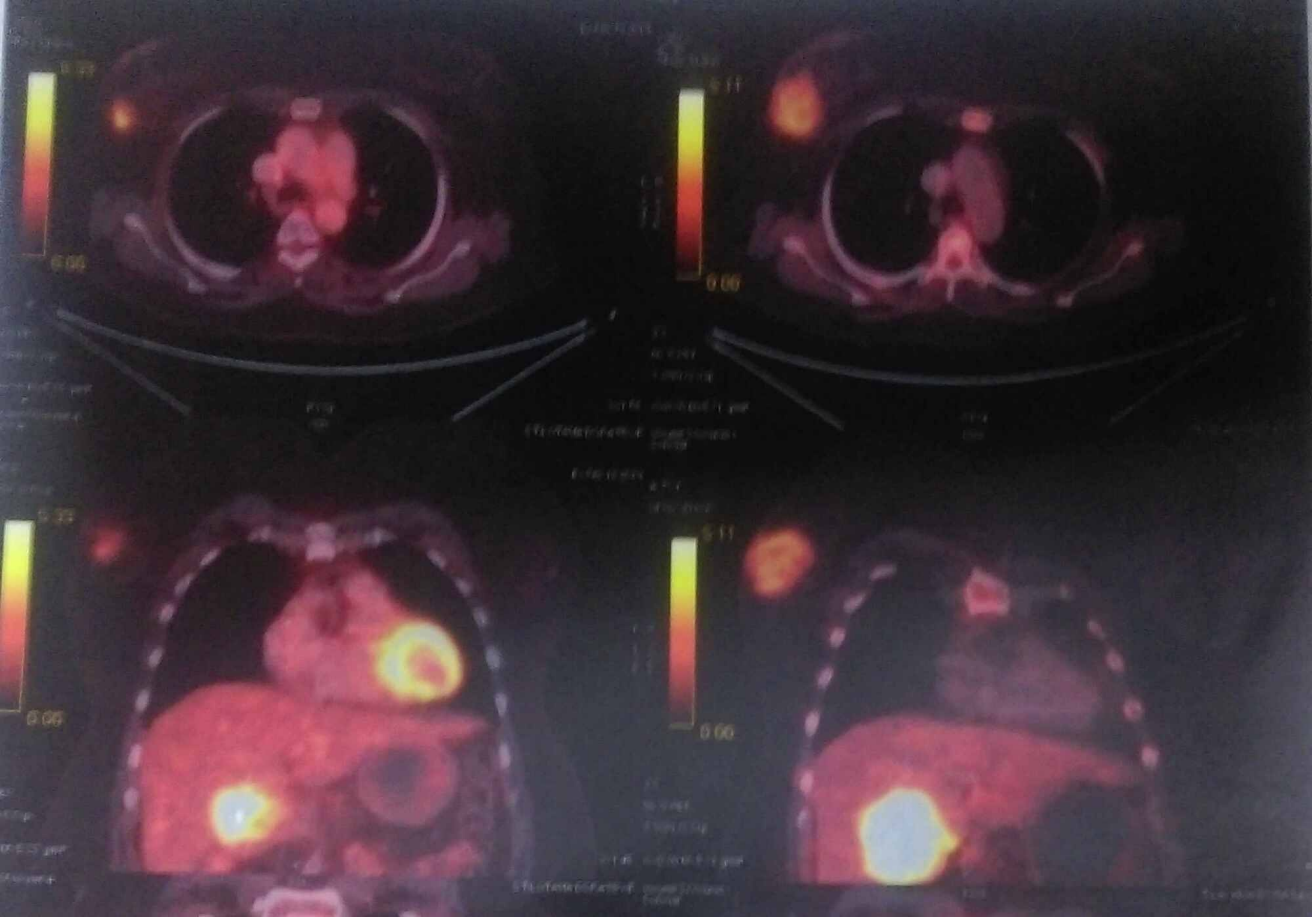
Whole-body PET-CT scan showing disease progression both in gallbladder fossa (size 4.9 x 6.3 x 6.1 cm, SUVmax = 9.86 and breast lump (size 6 x 4.6 cm, SUVmax = 3.37) compared to previous PET-CT scan PET-CT: positron emission tomography-computed tomography; SUVmax: maximum standard unit value

Palliative chemotherapy with nab-paclitaxel and gemcitabine was advised for three cycles. Interim evaluation with PET-CT scan showed disease progression (Figure 5).

Due to disease progression, the chemotherapy regimen was changed to nab-paclitaxel and carboplatin, but after taking two cycles of chemotherapy, she succumbed to the metastatic disease.

## Discussion

Gallbladder cancer (GBC) is the most common malignancy of the biliary tract, representing 80%-95% of biliary tract cancers worldwide. It is the sixth most common cancer among gastrointestinal cancers [1]. Although the etiology of the carcinoma of the gallbladder is obscure, cholelithiasis and chronic cholecystitis are the most frequent cause. GBC is three- to four-fold more prevalent among women than men, usually in the seventh decade of life [4].

GBC is aggressive cancer and has a poor prognosis. Five-year survival is only less than 10% [7-8]. Locoregional spread in GBC is more common than distant metastasis. The leading modes of dissemination in GBC are lymphatic, vascular, neural, intraperitoneal, and intraductal [9]. The common site of distant metastasis are the liver, abdominal lymph nodes, and peritoneum and rarely to the bowel, cervix, kidney, thyroid, and heart [10-11]. The lung is the most common site of extra-abdominal metastasis [12-13].

The incidence of breast metastasis from other primary sites accounts for only 0.5%-0.6% [14]. Breast metastasis is mostly seen from hematological malignancy, melanoma, and small cell carcinoma of the lung [15]. Metastasis to the breast from the gallbladder is significantly less and account for very few cases [6-16]. The hematogenous route is the most common mode of spread to the breast. Unlike primary breast malignancy, metastasis to the breast from other primaries are usually firm, well-circumscribed, and uninvolved skin. Mammography shows a lack of microcalcification. The upper outer quadrant is the most common location for involvement, mostly 62% [17].

Due to the rarity of the case, no definite consensus is there for proper diagnosis and management. Few studies show an average survival of 10-12 months, and with no intervention, survival is less than a month [18]. Gemcitabine-based chemotherapy is the most commonly used regimen. Aggressive management with systemic chemotherapy and surgery and radiation may extend overall survival to a few months. In our case, the patient underwent wide local excision of port site recurrence followed by two lines of palliative chemotherapy with gemcitabine with oxaliplatin and nab-paclitaxel with gemcitabine. Due to this aggressive multimodality treatment modality, our patient survived for 38 months from the diagnosis and around 25 months after breast metastasis.

## Conclusions

The incidence of breast metastasis in carcinoma gallbladder is scarce and a rare finding. Despite treating aggressively with chemotherapy, overall survival for these patients is inferior. We should consider multimodality treatment to improve survival.
